# Pulmonary Hydatid Cyst Rupture Associated With Albendazole Commencement: Case Report and Literature Review

**DOI:** 10.1155/crdi/8899087

**Published:** 2026-06-08

**Authors:** Thomas Ledger, Abiny Vignarajan, Ross Harvey, Lawrence Yuen, Rogan Lee, Bernard Hudson, Mark W. Douglas

**Affiliations:** ^1^ Centre for Infectious Diseases and Microbiology, Sydney Infectious Diseases Institute, The University of Sydney at Westmead Hospital, Westmead, New South Wales, Australia; ^2^ Department of Surgery, Westmead Hospital, Westmead, New South Wales, Australia, nsw.gov.au; ^3^ Parasitology Laboratory, NSW Health Pathology-ICPMR, Westmead Hospital, Westmead, New South Wales, Australia, nsw.gov.au; ^4^ School of Medical Sciences, Faculty of Medicine and Health, The University of Sydney, Westmead Hospital, Westmead, New South Wales, Australia, nsw.gov.au; ^5^ Department of Microbiology & Infectious Diseases, NSW Health Pathology, Royal North Shore Hospital, St Leonards, Sydney, 2065, New South Wales, Australia, nsw.gov.au; ^6^ Storr Liver Centre, The Westmead Institute for Medical Research and The University of Sydney, Westmead, New South Wales, Australia

**Keywords:** albendazole, *Echinococcus granulosus*, hydatid cyst rupture, hydatid disease

## Abstract

We report a 54‐year‐old immunocompetent female of Pakistani origin who presented with a 3 month history of abdominal fullness associated with hepatic and pulmonary cysts suggesting hydatid disease but negative *Echinococcus granulosus* serology. The hepatic cyst was resected, confirming hydatid disease, and perioperative albendazole was continued to treat the smaller pulmonary cyst. Three weeks after commencing albendazole, the patient developed acute chest pain due to rupture of the pulmonary cyst, with subsequent seroconversion for *Echinococcus granulosus*. A literature review identified four reports of ruptured pulmonary hydatid cysts during albendazole therapy, including a total of nine patients. Eight of 9 cases reported rupture of pulmonary cysts, all within 3 weeks of albendazole commencement, which was speculated to be due to decreased cuticular membrane pressures.

## 1. Introduction

The parasite *Echinococcus granulosus* is associated with cystic hydatid disease, following human ingestion of parasite eggs. Eggs arise from the definitive hosts in a two‐stage animal life cycle involving a definitive (typically dogs) and intermediate (typically livestock, including sheep, goats, camels, pigs, and others) host [1]. Cysts can arise in the lungs, liver, and other areas of the body, leading to symptoms either from mass effect or rupture [2]. Diagnosis is established with an often‐characteristic appearance on imaging and with serology, an important aid. Management strategies include antiparasitic therapy and surgical approaches, which may include cyst resection or percutaneous aspiration, injection, and reaspiration (PAIR), depending on the size and location of a cyst. Antiparasitic therapy in conjunction with resection or PAIR is recommended to reduce the risk of recurrence by past and recent guidelines [1, 2]. We describe in this case a patient with a cyst rupture associated with commencement of the antiparasitic agent albendazole. A literature review demonstrates this uncommonly reported adverse effect has been associated with albendazole commencement and draws attending to this important rare occurrence.

## 2. Case Presentation

We report a 54‐year‐old immunocompetent female of Pakistani origin presenting with 3 months of right upper quadrant abdominal pain and abdominal fullness. She had migrated to Australia 26 years previously and worked in childcare. Computed tomography suggested hepatic and pulmonary hydatid cysts, with a large cyst in segments VI and VII of the liver (90 × 70 × 110 mm) and a lobulated cyst in the right lower lobe of the lung (60 × 40 × 44 mm; Figure 1). *Echinococcus granulosus* (hydatid) serology was negative (VirClia Vircell, IgG index 0.14). A diagnosis of hydatid disease with intact hepatic and pulmonary cysts was made based on radiological findings, with the assumption of epidemiologically acquired disease prior to her migration from Pakistan, which had not been visited since the time of migration. No other clear exposure was identified in the history.

**Figure 1:**
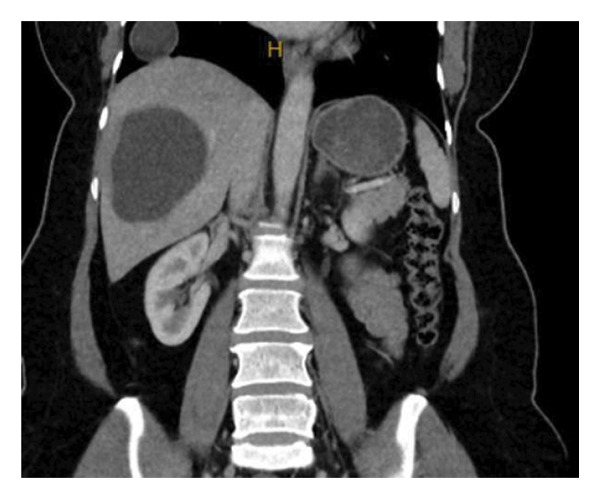
Coronal slice of computed tomography (CT) scan showing hydatid cysts, with a large cyst in segments VI and VII of the liver, and a lobulated cyst in the right lower lobe of the lung.

The symptomatic hepatic cyst was resected with an open segments VI and VII segmentectomy with no macroscopic intraoperative spillage. Protoscolesces were seen in the aspirate of the resected cyst, and margins were clear histologically. Albendazole 400 mg twice daily was commenced 7 days prior to the procedure and continued postoperatively, with a plan to continue for 3 months to sterilize the pulmonary cyst.

Three weeks after commencement of albendazole, the patient presented with right chest pain and reported coughing up viscous “grape skin”—like material. Imaging showed a right‐sided pleural effusion adjacent to her previous cyst, which was suspected to be a ruptured hydatid cyst. Praziquantel was commenced but discontinued due to severe nausea and vomiting associated with the first two doses. Amoxicillin and clavulanic acid 875/125 mg twice daily was commenced a 2 week period to cover for typical bacterial pathogens and the patient underwent a video‐assisted thoracoscopic (VATS) wedge resection of the right lower lobe with a 1 L saline washout. A macroscopically intact cyst with a small volume serous effusion adjacent was seen. Pleural fluid did not demonstrate protoscolesces on microscopy. *Echinococcus granulosus* serology performed 1 month later was positive (VirClia Vircell, IgG index 5.41), demonstrating seroconversion. The patient completed 3 months total of albendazole 400 mg BD.

## 3. Discussion

This patient received guideline‐based treatment, with surgery based on the size of her liver cyst and use of albendazole alone for the lung cyst [2]. This approach also remains concordant with a recent WHO guideline update [1]. The guideline describes two situations in which surgery is recommended: complicated liver cysts and pulmonary cysts > 5 cm. A PAIR approach remains recommended for many circumstances. This involves aspiration of cyst contents, injection of a protoscolicidal agent such as 20% ethanol, and re‐aspiration after 15–20 min. A “watch and wait” approach can be considered for stable, inactive, and small lesions [1].

In uncomplicated cysts (e.g., pulmonary or hepatic < 5 cm), the use of the benzimidazole albendazole alone is recommended. Albendazole is also recommended for use in conjunction with PAIR or open/laparoscopic surgical approaches to reduce the risk of recurrence. The highest quality evidence supporting the adjunctive use of albendazole and its preoperative use is a prospective randomized controlled trial of 64 patients [3]. This demonstrated a significant difference in disease recurrence between patients who received albendazole perioperatively (2 of 48; 4.16%) and those who did not (3 of 16; 18.75%) [3]. Albendazole, as compared with mebendazole, is the preferred benzimidazole due to increased rates of treatment success in a prospective trial of 51 patients. In this trial, success was determined by imaging findings of decreased cyst size and seen in 10 of 23 patients (43.5%) of those prescribed albendazole, as compared with 8 of 28 (28.6%) of those prescribed mebendazole for 6–24 months [4]. Albendazole is recommended to be given cautiously in chronic hepatic disease and in bone marrow suppressive states and to be avoided in early pregnancy [2].

Hydatid cyst rupture whist taking albendazole has been reported and is speculated to be mediated by decreased hydatid cyst cuticular tensile strength, increasing the risk of spontaneous rupture [5]. In one retrospective review, 14 of 176 cases of spontaneously rupturing cysts occurred whilst on albendazole, without characteristics specified [6]. A literature review was conducted, and we located four articles describing nine cases of hydatid cyst rupture whilst on albendazole that included details of cyst characteristics and time course (Table 1).

**TABLE 1:** Literature review of cyst rupture whilst on albendazole, with location and number of reported cases, and duration after commencement of albendazole.

Article (first author, year)	Location (*n*)	Duration (days)
Bulut, 2017 [7]	Hepatic (1)	1
Sheikhy, 2015 [8]	Pulmonary (1)	20
Kurkcuoglu, 2002 [9]	Pulmonary (1)	8
Koul, 2006 [10]	Pulmonary (6)	7, 8, 10, 10, 11, 13

Most reports of rupture report pulmonary lesions (8 of 9; 89%), all of which ruptured within the first 3 weeks of albendazole commencement and most within the first 2 weeks. It is suspected that the tensile strength of cysts is decreased after albendazole exposure, based on the observation that tensile strength in excised hydatid cysts was lower in cysts from patients exposed to albendazole, as measured in a prospective study of 44 patients, with strength measured using a tensiometer with a monofilament attached to either end of the membrane, pulling at increased pressure until cuticular rupture [5]. We speculate that pulmonary cysts may rupture more commonly than hepatic cysts because of variable intrapulmonary pressures associated with inspiration and expiration, as compared with stable intrahepatic pressures in the context of surrounding liver parenchyma.

In this case, seroconversion was associated with clinical and radiological features supportive of cyst rupture. Hydatid serology refers to antibodies against encysted antigens, generally those found in fluid, namely, antigen B, and antigen 5 [11]. Intact, calcified, and smaller cysts, and cysts in immunocompromised states such as HIV, may not expose antigens to antigen‐presenting cells and are associated with negative serology [11]. Ruptured cysts demonstrate a higher rate of positive serological findings than unruptured cysts, presumably by increasing the amount of antigen exposure [11]. This case proves an interesting example of the utility of serology to support the diagnosis of cyst rupture.

## 4. Conclusion

This case describes a 54‐year‐old immunocompetent female who experienced a ruptured hydatid pulmonary cyst whilst on albendazole 3 weeks after resection of a symptomatic hepatic cyst was resected, confirming hydatid disease, and perioperative albendazole was continued to treat the smaller pulmonary cyst. A literature review identified nine cases of cyst rupture whilst on albendazole, with eight of the nine cases being pulmonary cysts, which is speculated to be due to effects of albendazole on hydatid cyst cuticular membrane pressures. Albendazole is associated with hydatid cyst rupture, usually within 2 weeks of commencement, particularly with pulmonary cysts. Infectious disease physicians and microbiologists should be aware of this phenomenon.

## Author Contributions

All authors contributed to the writing of the manuscript.

Thomas Ledger contributed to the primary manuscript, review, and submission.

Abiny Vignarajan contributed to writing the case.

Ross Harvey contributed to the case discussion and review.

Lawrence Yuen contributed to the case discussion and review.

Mark W. Douglas provided significant input into the manuscript and review.

Rogan Lee contributed to the discussion and reviewing the case.

## Funding

Open access publishing facilitated by The University of Sydney, as part of the Wiley ‐ The University of Sydney agreement via the Council of Australasian University Librarians.

## Consent

Written consent was obtained from the patient.

## Conflicts of Interest

The authors declare no conflicts of interest.
